# The paradox of implementation: Tools without impact ‐ reflections from the global evidence summit

**DOI:** 10.1002/gin2.70010

**Published:** 2024-12-26

**Authors:** Laura Mora Moreo

**Affiliations:** ^1^ Department of Health Services Research and Policy London School of Hygiene and Tropical Medicine London UK

The Global Evidence Summit (GES2024), held in Prague from September 9 to 13, 2024, united scholars, healthcare professionals, and policymakers to tackle global health challenges. Among the diverse topics presented, implementation science stood out, emphasising collaboration to bridge evidence and practice gaps while fostering discussions on improving health systems and outcomes. Notably, many talks and posters focused on implementing clinical guideline recommendations—a critical and growing area of interest. However, a paradox remains: *whilst the development of implementation tools is on the rise, their actual influence on clinical outcomes remains largely unquantified*.

The GES2024 presented numerous presentations on digital technologies, decision‐support systems, and other advanced tools for disseminating and implementing clinical guidelines. For example, one exhibit showcased a mobile app designed to integrate palliative care guidelines into daily practice through a web‐based platform for healthcare professionals. Despite their popularity and high levels of user engagement, evidence of these innovations' effectiveness in improving patient outcomes was limited. Many implementation tools gauge success through superficial metrics like downloads or user engagement, which primarily reflect conceptual use,^1^ while these metrics indicate awareness or understanding, they rarely lead to the behavioural or process changes characteristic of instrumental use. For meaningful and sustained impact, such efforts must progress beyond these surrogate outcomes to measurable clinical outcomes that directly improve patient care or system efficiency, aligning with the goals of knowledge translation and implementation science. For example, the palliative care platform received over 100,000 visits in its first 6 months, but data on patient impact were lacking. Measuring app downloads is different from evaluating their effect on patient outcomes. While high engagement numbers suggest interest, they do not indicate whether the guidelines improved patient care or symptom management.

This raises a critical question: **Are implementers failing to recognise the importance of measuring the true impact of these tools, or are clinical guidelines recommendations inherently difficult to assess in real‐world practice?**

The literature emphasises that focusing only on engagement without assessing clinical outcomes undermines the main goal of guidelines—improving patient care. There is still a significant lack of information about the effective implementation of these recommendations. Despite existing frameworks, there is a shortage of evidence about real‐world application and effectiveness. Research shows a persistent gap between creating guidelines and putting them into practice, often worsened by inconsistent adaptation across different contexts. This underscores the need for strong research methods to determine whether guidelines genuinely improve clinical practice and patient outcomes rather than relying solely on their potential impact.^2^, ^3^, ^4^

Successful implementation of clinical tools requires more than availability; it demands integration into routine practice, alongside continuous evaluation. The lack of data on how well these tools are embedded in healthcare demonstrates the need for comprehensive assessments of implementation outcomes. Guideline recommendations are complex to implement, and the strategies should integrate several approaches. Technological tools can be useful, and their effectiveness has been proven in the past,^5^ but it's important to consider that they are only a small piece of the puzzle. The hindrances behind a good implementation process are varied and depend on the specific context targeted for implementation.^6^, ^7^

The discussions at the GES offered a platform to rethink how the implementation of recommendations should be evaluated. This editorial seeks to raise awareness that implementing recommendations extends beyond technical assistance systems. It emphasises the importance of considering the broader context, including local adaptation, sustainability, and real‐world outcomes, to ensure that guidelines effectively improve practice and patient care. The implementation process must be viewed as a dynamic, multifaceted endeavour rather than a purely technical task.

## AUTHOR CONTRIBUTIONS

I, Laura Alejandra Mora Moreo, confirm that I am the sole author of this manuscript. I made substantial contributions to the conception and interpretation of the work, drafted the manuscript, and revised it for important intellectual content.

## Data Availability

Data sharing is not applicable to this article as no datasets were generated or analysed during the current study.
